# Mapping the Healthiness of Community Food Environments and Their Association With the Socioeconomic Index for Areas (SEIFA) in the South Coast of NSW

**DOI:** 10.1002/hpja.70166

**Published:** 2026-02-24

**Authors:** Alemayehu Digssie Gebremariam, Katherine Kent, Chris Brennan‐Horley, Suzanne Pickles, Karen Charlton

**Affiliations:** ^1^ School of Medical, Indigenous and Health Sciences, Faculty of Science, Medicine and Health University of Wollongong Wollongong New South Wales Australia; ^2^ Department of Public Health College of Health Sciences, Debre Tabor University Debre Tabor Amhara Ethiopia; ^3^ School of Social Sciences, Faculty of Arts, Social Sciences and Humanities University of Wollongong Wollongong New South Wales Australia; ^4^ School of Health Sciences University of Newcastle Newcastle New South Wales Australia; ^5^ Hunter Medical Research Institute Newcastle New South Wales Australia

**Keywords:** community food environment, food environment score, food outlets, healthiness, socioeconomic index for areas

## Abstract

**Issue Addressed:**

The community food environment may influence consumers' dietary behaviours, particularly in socioeconomically disadvantaged areas. There is a paucity of evidence on the distribution of food outlets and their association with socioeconomic position in regional Australia. This study mapped the healthiness of food environments in the South Coast of NSW and examined associations with socioeconomic advantage and disadvantage.

**Methods:**

A cross‐sectional study using registered food outlet data, supplemented by Google Maps search, was conducted. The Food Environment Score (FES) was used to assign a three‐category healthiness score to food outlets, then categorised into a binary variable (unhealthy and healthy/less healthy). Multiple logistic regression was used to identify associations between food outlet types, administrative cluster, and the Index of Relative Socioeconomic Advantage and Disadvantage (IRSAD; Quintiles 1–5), using quintile 3 as the reference.

**Results:**

Of the 2321 identified food outlets, nearly half (*n* = 1040; 44.8%) were categorised as unhealthy, while only a small proportion (*n* = 345; 14.9%) were categorised as healthy. Advantaged areas (IRSAD quintile 5 and 4) had 40% less (AOR 0.60 [0.5, 0.8]) and 42% less (AOR 0.58 [0.4, 0.8]) unhealthy food outlets than IRSAD quintile 3 areas, respectively, after adjusting for the administrative cluster. No difference was found between the disadvantaged areas (IRSAD quintiles 1 and 2) and IRSAD quintile 3.

**Conclusions:**

In the South Coast of NSW, unhealthy food outlets dominated the community food environment, particularly in socioeconomically disadvantaged and middle‐income areas. This study extends the existing evidence in the selected states and metropolitan areas to regional Australia.

**So What?:**

Targeted interventions, including public policy and public health initiatives, are needed to increase access to healthy food outlets and restrict unhealthy options, particularly in middle and disadvantaged socioeconomic communities.

## 1 Introduction

An unhealthy diet is the leading preventable risk factor for diet‐related noncommunicable diseases, particularly among low socioeconomic groups [1, 2]. Unhealthy diets are a major public health concern in Australia, contributing to 27 500 preventable deaths annually [3]. In 2021–2023, nearly all Australian adults (96%) failed to meet recommended fruit and vegetable intake, particularly in regional and socioeconomically disadvantaged areas, while discretionary foods accounted for 38.6% of total energy intake [4, 5, 6]. In New South Wales (NSW), dietary risks remain high, with about 95.5% of adults failing to meet fruit and vegetable intake recommendations and 25.2% consuming alcohol in excess of guidelines [7].

Access to healthy food outlets through the community food environment directly shapes dietary behaviours, which, in turn, affects population health outcomes [8, 9]. The food environment represents the interface where consumers interact with the food system to obtain and consume food [10]. It encompasses a wide range of considerations, such as physical access to food, economic conditions that affect food affordability, policy and regulatory frameworks that influence food availability and acceptability and socio‐cultural influences such as social norms and media influence [11, 12]. Within this broader concept, community food environments are commonly defined by individuals' proximity to various types of food outlets or by the density and diversity of food outlets within a defined geographical area [12]. Globally and in Australia, several studies have shown an increase in the number of unhealthy food outlets, particularly in socioeconomically disadvantaged areas [13, 14, 15, 16, 17, 18, 19, 20]. However, evidence remains limited in some regions. Most Australian evidence on community food environments comes from a small number of states and metropolitan settings, with limited comparable data available for New South Wales, particularly outside Sydney. In addition, regulatory responses to unhealthy food environments continue to lag.

At the national level, Australia has introduced key policies to support healthier food environments, including the Health Star Rating system, GST exemption on fresh produce, and identified the role of community food environments in the National Preventive Health Strategy [21] and the National Obesity Strategy [22]. However, there are gaps remaining in the regulation of unhealthy foods, such as the implementation of taxes or levies on unhealthy food items, such as those high in sugar, salt, or fat, and there are no mandatory policies ensuring the provision of healthy foods in public and private workplaces [23]. While the existing policies set national direction, their impact depends on state and local implementation. This legislative gap in NSW is mirrored by a lack of region‐specific evidence on the healthiness of local food environments, which may be limiting councils' capacity to justify regulatory or planning interventions.

State governments regulate community food environments through public health legislation, which determines local councils' ability to act [24, 25]. The state public health acts of Victoria [26], Western Australia (WA) [27] and South Australia (SA) [28] empower local government areas to develop public health plans to prevent and control diet‐related noncommunicable diseases, including strategies across the food system. However, New South Wales (NSW) [29] lacks such provisions, preventing councils from regulating food retail environments or integrating food policies into urban planning. Revising NSW's act to include such provisions could align it with other states and enhance national food policy cohesion.

Some local governments have the potential to influence food environments through zoning laws, planning policies, and public health initiatives. According to an analysis of Australian local government policies on creating a healthy, sustainable, and equitable food system, local government areas in NSW and Victoria have taken actions to reduce food waste, provide food for disadvantaged groups, offer education on food and nutrition, encourage the production and marketing of fresh and healthy foods, and improve access to drinking water. However, there is a lack of restrictions on the promotion and opening of unhealthy food outlets [30, 31]. Without the support of state‐level mandates, local governments in NSW lack the tools to regulate unhealthy food environments. Addressing this gap by allowing local governments to bring a health lens to urban planning and providing them with the regulatory power to restrict unhealthy food outlets could significantly improve public health by promoting healthier food environments.

Improvements in community food environments are effective when informed by rigorous, local evidence, such as evidence generated through academic‐community partnerships [32]. Building local‐level evidence is crucial, as it allows local governments to directly tackle the specific challenges and opportunities unique to their communities [30]. In the context of community food environments, mapping the healthiness of food outlets and examining their association with the Index of Relative Socioeconomic Advantage and Disadvantage (IRSAD) is one important step that can help food system stakeholders, including local councils, to understand inequalities in food access. This study is part of a larger ARC Future Fellowship project aimed at creating a healthier, more sustainable, and more equitable food system in the Illawarra Shoalhaven region through people, partnerships and policy. Evidence on the distribution of food outlet types and their association with IRSAD remains limited for the South Coast region of NSW, Australia. Therefore, the aim of this study is to map the healthiness of the community food environments in the South Coast Region of NSW, Australia, and to examine whether the availability of healthy food outlets differs by area‐level Index of Socioeconomic Advantage and Disadvantage (IRSAD), as measured by Socioeconomic Index for Areas (SEIFA).

## 2 Methods

### 2.1 Study Area

The study setting is the South Coast of NSW, which stretches south from Sydney to the Victorian border, framed by the Pacific Ocean in the east. Overall, it covers 15, 778 km^2^ and is home to approximately 498 703 people [33]. The region contains six local government areas (LGA): Wollongong, Shellharbour, Kiama, Shoalhaven, Eurobodalla and Bega Valley. The total populations of the local government areas are as follows: Wollongong (214 564 residents), Shoalhaven (108531), Shellharbour (76271), Eurobodalla (40592), Bega Valley (35942) and Kiama (23074) [33]. The region encompasses a mix of urban, regional and rural areas spanning Modified Monash categories MM1 (metropolitan areas) to MM5 (small rural towns). Under the Australian Statistical Geographic Standard (ASGS) Remoteness Areas Classification, the six local governments included in the study areas are categorised as Major Cities, Inner Regional, or a combination of predominantly Inner Regional with Outer Regional. Specifically, Wollongong and Shellharbour are classified as Major Cities; Kiama as an Inner Regional; and Shoalhaven, Bega Valley and Eurobodalla as comprising both Inner Regional and Outer Regional areas [34]. In the local government areas of the South Coast of NSW, approximately 49.5%–57.7% of adults did not meet the recommended fruit intake in 2022 [35]. However, no region‐specific data is available for the South Coast of NSW. National data from the Australian Bureau of Statistics (ABS) show that in 2023–2024, about 38%–39% of dietary energy from food and non‐food alcoholic beverages sales came from discretionary foods. Snack foods, confectionery and cereal‐based products made up most of this energy [36].

This study provides important objective, place‐based evidence to inform the development of a regional food strategy that will address current community needs for a healthier, more sustainable and equitable food system. Mapping the community food environment is an important step required to guide policy and community‐led interventions aimed at improving access to healthy food.

### 2.2 Study Design

#### 2.2.1 Food Outlet Data

A desk‐based cross‐sectional study was conducted between July 2023 and February 2024 to map the healthiness of community food environments and identify their association with the IRSAD. A list of registered food outlets (Food outlet name and address) was obtained from the councils of each local government area in May and June 2023 and supplemented by a manual search for food outlets using keywords on Google Maps. The councils of each local government area were asked to email the list of food outlets, which were provided in spreadsheet format.

Each food outlet listed in the registry was first searched on Google Maps and matched with the corresponding address to confirm whether it was currently operating. When two or more outlets appeared with similar names, identification was based on the listed address. If a listed outlet did not appear on Google Maps, the suburb name was added to the search; if the outlet still could not be identified, it was classified as not currently operating. To identify additional food outlets not included in the registered list, further Google Maps searches were conducted using combinations of the suburb's name and food outlet type keywords including ‘supermarket’, ‘grocery’, ‘general store’, ‘fruit shop’, ‘vegetable shop’, ‘green grocer’, ‘fruiterer’, ‘butchery’, ‘poultry’, ‘fish shop’, ‘fast food’, ‘takeaway shop’, ‘restaurant’, ‘café’, ‘PUB’, ‘alcohol shop’, ‘bottle shop’, ‘club’, ‘hotel’, ‘convenience store’, ‘bakery’, ‘delicatessen’, and ‘cake shop’.

Information on outlet type, menu, opening hours, service mode and pictures was then reviewed to determine each outlet's primary offerings and categorise the outlets by type. Food outlet types were determined according to definitions established before data collection (Supporting Information S1). Thorough data cleaning was conducted, and duplicates and those not currently operating were removed from the list. Home businesses, school canteens, temporary food stalls, sports complexes with short opening hours, pharmacies, and other food outlets that are not open to the public (i.e., nursing homes, school canteens, home businesses and sports clubs) were excluded.

The Food Environment Score (FES) was used to assign a healthiness score to each food outlet based on a 20‐point scoring system ranging from −10 (least healthy) to +10 (most healthy), by matching the food outlet's type [37]. Each physical food outlet was treated as a single analytical unit and assigned one FES score based on its dominant food offering. Where outlets sold a range of food products, classification was based on the outlet's primary function. The classification and healthiness score rating were performed by two independent raters, and differences in the rating were resolved by discussion.

Food outlets were categorised into 18 categories based on the FES and were then reclassified into 10 categories as follows for ease of reporting (Table 1).

**TABLE 1:** Categories of food outlets.

New classification	FES category	Food environment score
Fruiterer and greengrocer	Fruiterer and greengrocer	+10
Fish shop	Fish shop	+10
Butchery	Butchery	+9
Sandwich shop	Sandwich shop	+5
Supermarket	Major supermarket, Minor supermarket and specialty food store (core)	+5
Restaurant/café	Restaurant/café (franchise) and Restaurant/café (local)	0
Bakery/cake shop	Bakery/cake shop and delicatessen	0
Convenience store	Convenience store and Service station convenience	−10 to −5
Fast‐food outlet	Takeaway (franchise), Takeaway (local) and Specialty food store (extra)	−10 to −8
Alcohol outlets	Liquor‐selling stores and Pubs	−10 to −8

Healthiness ratings were classified into three categories: healthy (FES +5 to +10); less healthy (FES −4 to +4); and unhealthy (FES −10 to −5), which were then categorised into binary as either unhealthy or healthy/less healthy for the analysis [37]. The ratio of unhealthy to healthy/less healthy food outlets was also calculated.

#### 2.2.2 Geographic Areas

Local Government Areas (LGAs) are administrative regions governed by local councils, with boundaries determined by state or territory governments to facilitate local service delivery and administrative oversight [38]. Statistical Area Level 2 (SA2) regions are medium‐sized geographic units defined by the ABS under the ASGS to support standardised census data analysis; SA2s are composed of Statistical Area Level 1 (SA1) units to represent communities with shared social and economic characteristics, typically with populations of 3000 to 25 000. Suburbs and Localities (SALs) are officially designated areas defined by state authorities to strengthen community identity and urban planning through clearly defined public boundaries; SALs are generally smaller than SA2s, with SA2s often aligning with a single suburb in urban settings, while in rural areas, multiple SALs are commonly aggregated within a single SA2 [39].

#### 2.2.3 Socioeconomic Index for Areas (SEIFA)

The Socioeconomic Index for Areas (SEIFA) is a set of indices developed by the Australian Bureau of Statistics (ABS) to measure the relative socioeconomic status of different geographic areas across Australia. It is based on population characteristics such as income, education, employment, and access to resources. SEIFA includes four indexes: the Index of Relative Socioeconomic Advantage and Disadvantage (IRSAD), the Index of Relative Socioeconomic Disadvantage (IRSD), the Index of Economic Resource (IER) and the Index of Education and Occupation (IEO) [40]. IRSAD was found to be the best area‐level socioeconomic indicator for this study because it measures both relative advantage and disadvantage across a broad range of socioeconomic characteristics, including income, education, employment, occupation, housing, and access to material and social resources, factors important to the community food environments [41]. It is categorised into quintiles. Quintile 5 represents the most advantaged areas, while quintile 1 represents the least advantaged [40]. Data on IRSAD was extracted from the 2021 ABS census data using a predesigned data extraction sheet.

### 2.3 Data Analysis

Frequencies and percentages were used to describe the types of food outlets and their healthiness categories. First, binary logistic regression was conducted to examine the association between IRSAD at the SAL level, administrative clusters, and the healthiness of food outlets. Covariates were incorporated into the regression analysis using a staged modelling approach. Variables with *p*‐values < 0.2 were included in the multiple logistic regression. Multiple logistic regression was used to identify the independent effect of administrative clusters and IRSAD at the SAL level on the healthiness of food outlets. IRSAD quintile 3 (the middle socioeconomic group) was used as the reference category to enable comparison of both more disadvantaged (quintiles 1–2) and more advantaged (quintiles 4–5) areas against the socioeconomic average; sensitivity analyses using IRSAD quintile 1 as the reference (Supporting Information S2). Shoalhaven was used as the reference region in regression analyses because descriptive analyses indicated it had the healthiest food environment among the regions studied.

Local government areas were grouped into regions based on administrative clusters: Illawarra (comprising Wollongong, Shellharbour and Kiama), Shoalhaven, and Sapphire Coast (including Eurobodalla and Bega Valley). The data were analysed using SPSS version 29.0 for Windows.

Graphical maps of the IRSAD and the tertiles of the ratio of unhealthy to healthy/less healthy food outlets at SA2 were created based on the ABS (2021) census SA2 shapefile, using QGIS desktop version 3.24.1. Mappings were conducted at the SA2 level to capture community‐level variation in food environments with greater spatial resolution than SA3 or LGA‐level units. For mapping purposes, the ratio of unhealthy to healthy/less healthy food outlets was ranked into three categories and presented as tertiles.

## 3 Results

### 3.1 Distribution of Types of Food Outlets in the South Coast of NSW, 2024

Overall, there were 2321 food outlets operating in the South Coast region of NSW. Of these, the most common type was restaurant/café (*n* = 817; 35.2%), followed by fast‐food outlets (614; 26.5%) and alcohol outlets (256; 11%). Supermarkets (*n* = 194) accounted for 8.4% of all food outlets. The least common food outlets were fruiterers/greengrocers and fishmongers, each contributing less than 1% each (Table 2).

**TABLE 2:** The distribution of type of food outlets by local government area, 2024.

Food outlets type	Local government areas						
	Wollongong *N* (%)	Shoalhaven *N* (%)	Shellharbour *N* (%)	Kiama *N* (%)	Bega Valley *N* (%)	Eurobodalla *N* (%)	Total *N* (%)
Fast‐food outlets	309 (30.8)	90 (18.6)	93 (34.2)	30 (18.8)	35 (19.3)	57 (25.6)	614 (26.5)
Restaurant/Café	350 (34.9)	186 (38.5)	82 (30.1)	71 (44.4)	57 (31.5)	71 (31.8)	817 (35.2)
Alcohol outlets	98 (9.8)	63 (13.0)	18 (6.6)	20 (12.5)	28 (15.5)	29 (13.0)	256 (11.0)
Supermarket	76 (7.6)	45 (9.3)	21 (7.7)	8 (5.0)	21 (11.6)	23 (10.3)	194 (8.4)
Convenience store	76 (7.6)	19 (3.9)	22 (8.1)	12 (7.5)	21 (11.6)	20 (9.0)	170 (7.3)
Bakery/Cake shop	38 (3.8)	38 (7.9)	14 (5.1)	8 (5.0)	9 (5.0)	12 (5.4)	119 (5.1)
Butchery	13 (1.3)	20 (4.1)	9 (3.30)	4 (2.5)	7 (3.9)	7 (3.1)	60 (2.6)
Sandwich shop	30 (3.0)	9 (1.9)	10 (3.7)	5 (3.1)	1 (0.6)	2 (0.9)	57 (2.5)
Fishmonger	5 (0.5)	8 (1.7)	1 (0.4)	2 (1.3)	1 (0.6)	1 (0.4)	18 (0.8)
Fruiterer and greengrocer	7 (0.7)	5 (1.0)	2 (0.7)	0 (0)	1 (0.6)	1 (0.4)	16 (0.7)
Total	1002 (100)	483 (100)	272 (100)	160 (100)	181 (100)	223 (100)	2321 (100)
Total population	214 564 (43.0)	108 531 (21.8)	76 271 (15.3)	23 074 (4.6)	35 942 (7.2)	40 594 (8.1)	498 974 (100)

Of the total 2321 food outlets across the six LGAs, Wollongong LGA had the largest proportion of food outlets (*n* = 1002), of which the most common food outlets were restaurants/cafés (350; 34.9%), followed by fast‐food outlets (309; 30.8%). Shoalhaven LGA had the second largest proportion (483), followed by Kiama (160), Bega Valley Shire (181) and Eurobodalla (223). In terms of total population, Wollongong had the largest population, comprising 214 564 residents (43.0% of the study population), followed by Shoalhaven with 108 531 residents (21.8%) and Shellharbour with 76 271 residents (15.3%). Kiama had the smallest population, with 23 074 residents (4.6%) (Table 2).

### 3.2 Healthiness of Food Outlets

**FIGURE 1:**
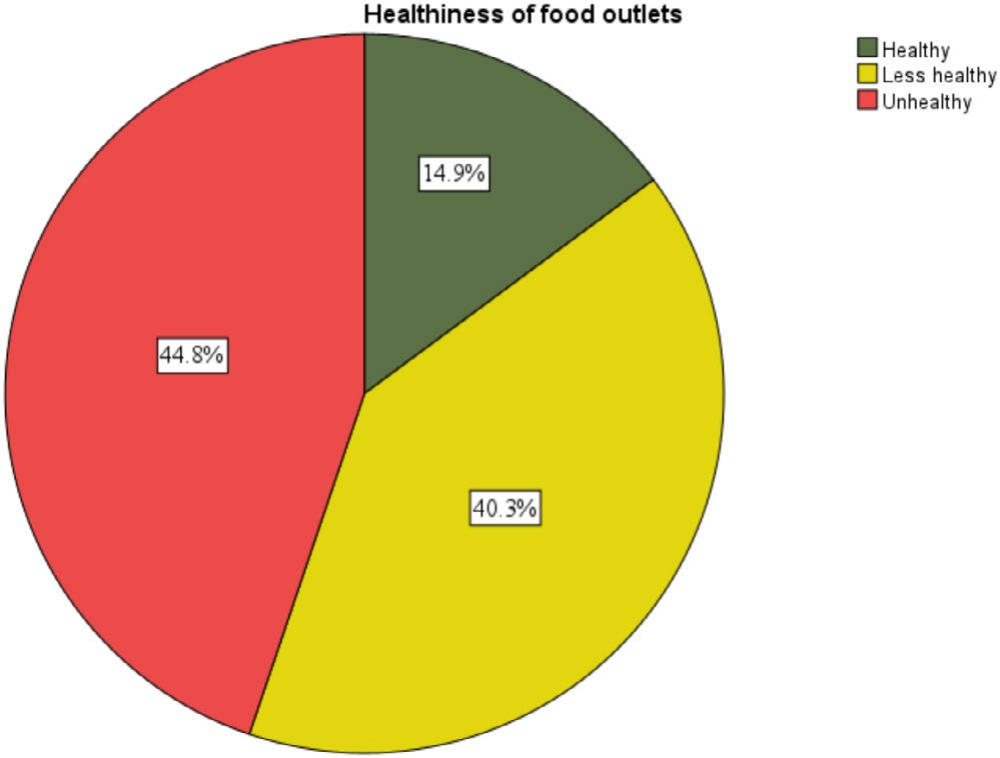
Healthiness rating of food outlets in the South Coast of NSW, 2024.

Of the 2321 food outlets, nearly half (*n* = 1040; 44.8%) were categorised as ‘unhealthy’, compared to only 14.9% (*n* = 345) categorised as ‘healthy’ and the remaining ‘less healthy’ (40.3%) (Figure 1).

### 3.3 Distribution of the Healthiness of Food Outlets Across Local Government Areas

**FIGURE 2:**
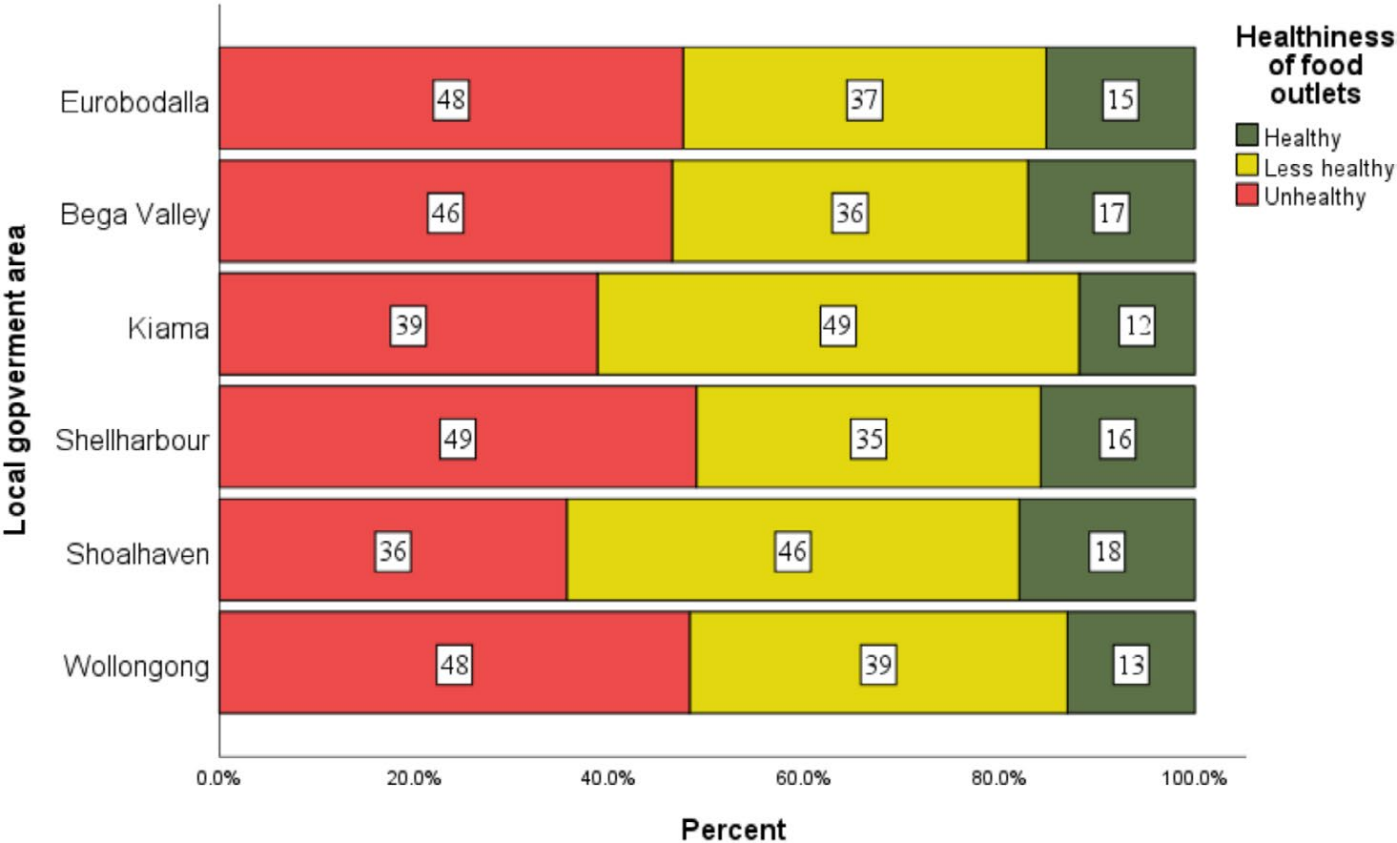
Distribution of healthiness rating of food outlets across Local Government Areas in the South Coast NSW, 2024.

Shellharbour and Wollongong had the highest proportion of unhealthy food outlets (48.9% and 48.2%, respectively) while Kiama had the largest proportion of less healthy outlets (49.4%), and Shoalhaven had the highest proportion of healthy outlets (18.0%). Overall, unhealthy outlets were the majority in most local government areas, followed by less healthy ones (Figure 2).

### 3.4 Healthiness of Food Outlets by Regions and Socioeconomic Index for Areas

Disparities in the healthiness of food outlets were observed across regions and SEIFA at SAL levels. Illawarra had 87% more unhealthy food outlets than Shoalhaven, as compared to healthy and less healthy food outlets (AOR 1.87 (95% CI: 1.5, 2.3)), while Sapphire Coast had 46% more unhealthy food outlets than Shoalhaven, as compared to healthy and less healthy food outlets (AOR 1.46 (1.1, 1.9)) (Table 3). Index of Relative Socioeconomic Advantage and Disadvantage (IRSAD) quintile 5 (indicating the most advantaged) had 40% less unhealthy food outlets, as compared to healthy/less healthy outlets, than IRSAD quintile 3 (AOR 0.60 (0.5, 0.8)). IRSAD quintile 4 had 42% fewer unhealthy food outlets compared to healthy and less healthy food outlets than IRSAD quintile 3 (AOR 0.58 (0.4, 0.8)). There were no statistically significant differences in healthiness categories of food outlets between IRSAD quintiles 1 and 2 (most disadvantaged) and quintile 3 (Table 3).

**TABLE 3:** Association between the number of food outlets by healthiness across regions and Index of Socioeconomic Advantage and Disadvantage (IRSAD) at the suburb and localities level, 2024.

		Number of healthy and less healthy outlets *n* (%)	Number of unhealthy outlets *n* (%)	COR (95% CI)^a^	AOR (95% CI)^b^
Administrative cluster	Illawarra	756 (59.0)	678 (65.2)	**1.62 (1.31, 2.00)**	**1.87 (1.49, 2.34)**
	Sapphire Coast	214 (16.7)	190 (18.3)	**1.61 (1.23, 2.10)**	**1.46 (1.11, 1.92)**
	Shoalhaven	311 (24.3)	172 (16.5)	1	1
Index of Socioeconomic Advantage and Disadvantage (IRSAD)	1	386 (30.1)	345 (33.2)	0.86 (0.67, 1.12)	0.97 (0.74, 1.27)
	2	192 (15.0)	171 (16.4)	0.86 (0.64, 1.16)	0.90 (0.66, 1.21)
	3	164 (12.8)	170 (16.3)	1	1
	4	249 (19.4)	170 (16.3)	**0.61 (0.46, 0.81)**	**0.58 (0.44, 0.77)**
	5	290 (22.4)	184 (17.7)	**0.66 (0.50, 0.88)**	**0.60 (0.45, 0.81)**
Total		1284 (100)	1037 (100)		

^a^ COR: crude odds ratio.

^b^ AOR: adjusted odds ratio; the two independent variables were the Index of Relative Socioeconomic Advantage and Disadvantage (IRSAD) at the suburb and localities level and administrative cluster, which was formed by combining the local government areas; administrative cluster adjusted to IRSAD; IRSAD adjusted to administrative cluster.

### 3.5 Mapping of the Healthiness of Food Outlets and IRSAD at the Statistical Area Level 2

**FIGURE 3:**
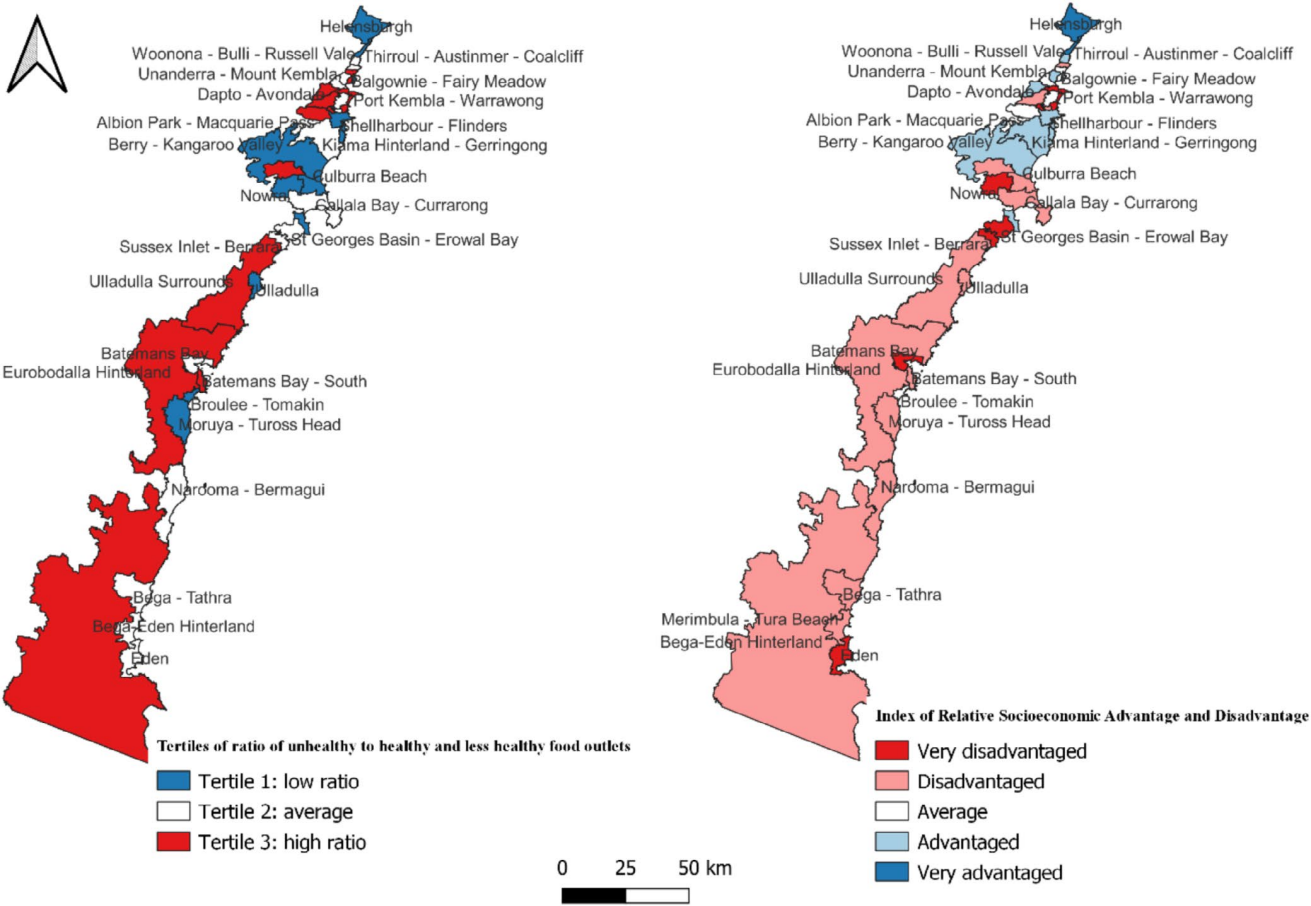
Map of tertiles of the ratio of unhealthy to healthy/less healthy food outlets and quintiles of IRSAD at SA2 in South Coast of NSW, 2024.

Figure 3 presents tertiles of the ratio of unhealthy to healthy/less healthy food outlets mapped alongside quintiles of the Index of Relative Socioeconomic Advantage and Disadvantage (IRSAD) at the SA2 (Figure 3). Visual comparison of the maps indicates that several SA2s, including Port Kembla‐Warrawong, Albion Park Rail, Berkeley‐Lake Heights‐Cringila, Warilla, Dapto‐Avondale, North Nowra‐Bomaderry, Batemans Bay‐South and Eurobodalla Hinterland, fall within the highest tertile of the ratio of unhealthy to healthy/less healthy outlets and are also characterised by lower socioeconomic advantage and disadvantage. In contrast, SA2s such as Helensburgh, Thirroul‐Austinmer‐Coalcliff, Berry‐Kangaroo Valley, Huskisson‐Vincentia, Kiama, Kiama Downs‐Minnamurra, and Shellharbour‐Flinders are in the lowest tertile of the ratio and are among the more socioeconomically advantaged and disadvantaged areas. Overall, the mapped distributions suggest a spatial pattern whereby a higher ratio of unhealthy to healthy/less healthy food outlets tends to co‐occur with lower socioeconomic position at the SA2 level. This observation suggests a potential spatial correlation between the ratio of unhealthy to healthy/less healthy food outlets and the IRSAD at the SA2 level.

## 4 Discussion

The purpose of this study was to assess the healthiness of community food environments across the South Coast of NSW, examine disparities in food access, and explore associations with socioeconomic factors to inform policy and community interventions. This study complements previous findings from Penrith, NSW [13], Victoria [14, 15], Perth, WA [19] and New Zealand [42] demonstrating an abundance of unhealthy food outlets, particularly in areas of higher disadvantage and extends this evidence to the regional context in NSW. Our study joins the growing body of evidence and underscores a need for further advocacy to improve the type and number of food outlets and food retailers in a geographical area.

Aligned with other research findings, our data demonstrates that unhealthy food outlets dominate the community food environment across the South Coast of NSW, while healthy food outlets account for only a small proportion in each region. Indeed, longitudinal studies conducted in Perth (2004–2011) [16] and Greater Melbourne (2008–2016) [15], have demonstrated that the number of unhealthy food outlets has increased in these Australian regions over time, showing a concerning trend in the proliferation of unhealthy food outlets in Australia. Such changes in the built environment are also coupled with an increase in the availability of unhealthy food options through online delivery platforms. For example, a recent study by Partridge et al. found that approximately 73% of food outlets available through online food delivery in Australia and New Zealand were unhealthy [17] which aligns with research from Victoria [14] and Perth, WA [19]. A key consideration while interpreting these findings is that analyses based on outlet classification, count, and spatial distribution often overlook sales volume. Supermarkets, usually classified as healthier food outlets, are less densely spread but account for a large share of total food purchases due to their size and wide offerings. Conversely, restaurants, cafes, takeaways and convenience stores are more common but contribute less to total food sales. From 2019 to 2023, supermarkets and grocery stores made up over 60% of food and drink sales in Australia, while restaurants, cafes and takeaways accounted for 21%–28%, despite being more numerous [43]. Supermarkets offer access to healthy foods but are also a major source of unhealthy products. Evidence from Brazil shows that most ultra‐processed food purchases occur in supermarkets [44]. These findings highlight the complexity of food environments and emphasise the need to consider outlet density alongside purchasing patterns, urbanisation, and the influence of multinational food corporations and marketing practices [45, 46].

Of concern, our study shows that socioeconomically disadvantaged and middle socioeconomic areas had a higher proportion of unhealthy food outlets as compared to socioeconomically advantaged areas. This finding aligns with the studies conducted on food outlets available through online food delivery platforms in Victoria, Australia [14] and Auckland, New Zeeland [17]. Similarly, a study conducted in Perth found that neighbourhoods in socioeconomically disadvantaged areas had a greater proximity to unhealthy food outlets as compared to neighbourhoods in socioeconomically advantaged areas [13]. Our research extends these findings to a large regional setting, reinforcing concerns about the emergence of ‘food swamps’, which are areas oversaturated with unhealthy food options. Several factors drive this trend. Independent takeaway outlets, convenience stores and alcohol outlets have low establishment and operating costs and achieve high profit margins, making them viable in socioeconomically disadvantaged neighbourhoods [47, 48]. Alcohol outlets are often located in such areas and can significantly impact the local food environments [49, 50, 51, 52]. However, it remains unclear whether higher demand for unhealthy food drives this trend or if retailers deliberately target low‐income areas due to lower costs and less competition from healthy food outlets [53, 54]. This warrants further investigation.

Regardless, unhealthy food environments pose a serious public health challenge in high‐income countries, contributing to inequitable access to healthy food and increasing the risk of diet‐related noncommunicable diseases such as obesity, diabetes, and cardiovascular disease [54, 55, 56]. Positively, effective policy responses and public health initiatives at various levels have been shown to improve the healthiness of food environments and reduce their impact on diet‐related noncommunicable diseases [57]. For example, the International Network for Food and Obesity/Noncommunicable Diseases Research, Monitoring and Action Support (INFORMAS) has been working to improve food environments and implementation of policies and actions to prevent obesity and diet‐related noncommunicable diseases at national and local levels [58]. INFOMAS has developed a tool to evaluate and monitor food environments (Food‐EPI), which has been adopted in more than 50 countries, including Australia [59]. In addition, the Milan Urban Food Policy Pact (MUFPP) [60] and Barilla Foundation's Food Sustainability Index [61] also undertook similar initiatives to monitor the food environments and support local governments based on good practice [62]. For example, a scoping review that identified food policies implemented in the 199 signatory cities of the MUFPP has demonstrated that urban local governments are implementing policies that consider multiple phases of the food supply chain to facilitate population‐wide uptake of healthy and sustainable diet‐related practices. The most common strategies involved food procurement within public facilities (44%) and establishing guidelines for school‐feeding programs (33%). The review concluded that opportunities exist for local governments to leverage the dual benefits to human and planetary health of policy actions, such as those which discourage the overconsumption of food, including less meat consumption and the regulation of ultra‐processed foods [63].

While Australia lags behind in local‐level food regulation, international case studies from the USA, Canda and the UK demonstrate how local governments can reshape food environments through policy and planning, which local governments could trial within the NSW South Coast Region. For instance, the establishment of initiatives such as ‘healthy corner stores’ in Washington, D.C, has increased the availability of healthy food items through existing food outlets where the sale of unhealthy foods predominates, demonstrating sustainable access to healthy foods among low‐income communities in areas without supermarkets [64]. Other research has reported that the establishment of healthy food outlets such as ‘nonprofit grocery stores’ in Philadelphia, Pennsylvania, and food cooperatives in Olympia, Washington, D.C, can counteract the inequalities of healthy food access [65, 66]. Local governments have also worked to implement organisational procurement policies in council owned and run businesses (such as hospitals and schools) in Los Angeles, California, which have improved access to healthy food for low‐income communities and enabled consumers to get cost‐effective foods [67]. Beyond this, the Farm to School initiative in Vermont has trailed a new model that improves children's nutrition and strengthens the local agricultural economy by connecting food producers with local schools and incorporating nutrition education [68]. Furthermore, permitting and licensing policy in Minneapolis, Minnesota, and land use and zoning ordinances in Boston, Massachusetts, have worked to enhance the availability of community gardens and farmers' markets, which have ultimately enhanced healthy eating behaviours in those communities [69]. In Canada, community‐supported agriculture, mobile markets and urban agriculture initiatives have improved food access and affordability and have been shown to enhance dietary outcomes at the population level [70].

In Australia, interventions to improve the healthiness of food environments remain in their early stages, with inconsistent implementation across states and limited focus on socioeconomic inequalities. Some local governments in Victoria, Western Australia and South Australia [59] have leveraged frameworks such as the Food Environment Policy Index (Food‐EPI) and collaborated with advocacy coalitions such as the Sustain—the Australian Food Network, to develop and implement strategies, with varying success. These methods help identify policy gaps and support local action, but they often miss how socioeconomic disadvantage affects the distribution of food outlets. For example, in Victoria, the City of Greater Bendigo developed the Bendigo Regional Food System Strategy 2020–2030, which includes zoning regulations to limit unhealthy food outlets near schools and support healthier food environments through collaboration and community engagement [71]. In addition, as noted by Dangerfield et al., there is a significant opportunity to integrate public health and wellbeing plans into Australian regional local government planning [72], with strong community support for policy intervention aimed at improving healthy food environments in public facilities [73]. However, many local governments across NSW and Victoria have cited a lack of community interest as a barrier to developing comprehensive food system strategies, highlighting a disconnect between public interest and government perceptions [74].

This study has a number of strengths, including populating a database of registered food outlets obtained from each local government council in the catchment area, validated by a search using Google Maps at the suburb and locality levels to ensure all available food outlets in the study area were included, and those that were no longer functional were excluded. Thorough data cleaning was implemented using longitude and latitude coordinates of outlets listed in the database. Despite this fact‐checking process, the accuracy of Google Maps is dependent on updates made by the owners and might not always reflect the real‐time status. A validated food environment score [37] was used to evaluate the healthiness of food outlets, with ratings conducted independently by two authors to ensure reliability. A reliable and widely accepted socioeconomic indicator (SEIFA) is used to assess the socioeconomic status of areas. Despite these strengths, this study has several limitations. First, the desk‐based nature of the assessments may have resulted in misclassification of the food outlets. Although Google Maps was used to validate food outlet data, its accuracy depends on business updates and may not reflect the real‐time changes. Future desk‐based assessments could be enhanced through field validation of a subsample of outlets to improve classification accuracy. Second, food outlet classification was based on a validated scoring system (Food Environment Score) that categorises outlets as healthy, less healthy, or unhealthy; however, this approach did not capture variations in the specific types and amounts of food sold within each outlet, which could be explored in future research. While the Food Environment Score was appropriate for this mixed urban–regional study, other food environment assessment tools have been developed for rural contexts and could be explored in future research focused specifically on rural food retail environments. For regression analysis, healthy and less healthy outlets were combined into a single category to improve interpretability and model stability; however, this approach may have introduced misclassification bias by obscuring differences in outlet types. In addition, although the food outlet assessment was conducted independently by two researchers, inter‐rater reliability was not formally captured. The regression model was not adjusted for factors such as population size and geographic size, which may have resulted in residual confounding. Finally, the study focused on the presence of food outlets and did not consider other influential factors, such as the proportion of sales volume in each food outlet, individual‐level affordability, accessibility, or online food delivery services, which influence food choices. Future research should explore longitudinal trends, the impact of digital food environments, and policy interventions to improve food access.

Recommendations

- Local councils, in collaboration with public health initiatives, should design short‐term and long‐term strategies to promote healthy food outlets and prohibit unhealthy options, particularly in socioeconomically disadvantaged areas.
- Local policymakers (state and federal), in collaboration with local councils, should work to develop a food strategy that fosters a healthier and equitable food system by encouraging the availability of healthy food outlets while limiting unhealthy options.
- Further research is required to understand consumers' perceptions of the community food environments, particularly in regional and socioeconomically disadvantaged areas.

## 5 Conclusion

This study is the first to highlight the abundance of unhealthy food outlets and significant disparities in the healthiness of food environments across the South Coast of NSW, with socioeconomically disadvantaged and middle‐income areas having a higher proportion of unhealthy food outlets compared to more advantaged areas. This study extends the existing evidence in the selected states and metropolitan areas to regional Australia. Addressing these disparities requires coordinated action across local, state and national levels, with stronger policy frameworks, urban planning regulations, and public health initiatives to support healthier food environments. Findings from this study will be critical in advocating for local councils to prioritise food issues in their strategy documents, considering zoning laws, incentives for healthy food retailers and community‐driven strategies that actively reshape the food environment. Further, this work aims to bolster advocacy efforts of local governments in navigating state and federal‐level legislative constraints, aiming to create a healthier, sustainable, and equitable food system that contributes to improving public health outcomes.

## Author Contributions

A.D.G., K.K., and K.C. designed the study. A.D.G. and S.P. collected and cleaned data with support from K.K., C.B., and K.C. A.D.G. analysed the data and drafted the initial version of the manuscript with support from K.K. and K.C. K.K. and K.C. reviewed and provided feedback on the subsequent version of the manuscript. All the authors read and approved the final manuscript.

## Funding

The authors have nothing to report.

## Ethics Statement

The study used publicly available data and does not require ethical clearance.

## Conflicts of Interest

The authors declare no conflicts of interest.

## Supporting information


**Supporting Information: S1.** Operational definitions of food outlet type.
**Supporting Information: S2** Table 2: Association between healthiness of food outlets across regions and Index of Socioeconomic Advantage and Disadvantage (IRSAD) at the suburb and localities level, 2024.

## Data Availability

The data that support the findings of this study are available from the corresponding author upon reasonable request.
