# Supplementary material for: Mapping the Healthiness of Community Food Environments and Their Association With the Socioeconomic Index for Areas (SEIFA) in the South Coast of NSW
Source: Health Promot J Austr. 2026 Feb 24;37(2):e70166. doi: 10.1002/hpja.70166 (PMC12930234; doi:10.1002/hpja.70166)
Supplement: Supplementary file 1 — Supporting Information: S1. Operational definitions of food outlet type. Supporting Information: S2 Table 2: Association between healthiness of food outlets across regions and Index of Socioeconomic Advantage and Disadvantage (IRSAD) at the suburb and localities level, 2024. [file HPJA-37-0-s001.docx]

Supplementary material 1: Operational definitions of food outlet type

Food outlets were categorised into mutually exclusive categories based on their primary food offerings and mode of service.

1. Major supermarket: large retail stores offering a wide range of food and beverage products, including fresh fruit and vegetables, meat, grains, dairy products, ready-to-eat foods, and discretionary foods. It may include a butcher or a baker. Usually have five or more checkouts and a floor area of over 1000 square metres. E.g. Woolworths, Coles, ALDI, etc.
2. Minor supermarket: a retail store selling groceries (fresh foods, canned and packaged foods, dry goods) of non-specialised (conventional) food lines. Usually has four or fewer checkouts and a floor area under 1000 square metres. E.g., an independent grocer or supermarket.
3. Take-away local: food outlets mainly selling ready-to-eat takeaway meals and snacks, with no table service. Customers can eat on-site, take away, or have their food delivered. These shops are usually independently owned, and common items include kebabs, burgers, pizzas, fried foods, and fries, such as kebab and fish & chips shops.
4. Take-away franchise: food outlets primarily focus on preparing and selling ready-to-eat meals and snacks. They do not offer table service; customers can eat on the premises, take food away, or have it delivered. These shops are usually part of a franchise or chain, selling food in specialised packaging, such as McDonald's, KFC, Domino's, etc.
5. Restaurant/café local: food outlets mainly focus on preparing and selling meals, hot drinks, and snacks for customers to eat on site. They typically offer freshly made foods tailored to individual orders. These establishments provide table service and often have takeaway options as well. Examples include culturally themed restaurants or cafés, such as Mexican, Thai, or Chinese, that are not primarily takeaway outlets.
6. Restaurant/café franchise: Food outlets mainly focus on preparing and selling meals, hot drinks, and snacks for on-site consumption with table service. They may also provide takeaway options. These establishments are often part of a franchise or chain, such as Guzman y Gomez, Oporto, and Espresso Bar.
7. Bakery/Cake shops: retail stores primarily offering bread, cakes, pastries, and other baked goods
8. Delicatessen mainly offers specialty packaged or fresh products such as cured meats, sausages, pickled vegetables, dips, bread, and olives. It might also serve dine-in meals.
9. Convenience stores are small retail outlets that mainly sell packaged and ready-to-eat foods, such as sweetened beverages and other discretionary items.
10. A service station convenience store is a retail outlet that combines fuel sales with quick access to drinks, snacks, basic groceries, and tobacco products.
11. Specialty store extra: primarily sells foods like ice-creams, doughnuts, waffles, cakes, and similar items, which are considered extra foods.
12. Speciality store core: Primarily focused on selling a limited range of specialised foods, such as whole foods, spices, cereals, grains, etc., which can be considered core foods. E.g. Indian grocery stores
13. Fruit and vegetable shop (fruiterers/greengrocers): retail establishments primarily engaged in the sale of fruits and vegetables.
14. Fish shop: specialty outlets selling mainly fresh and minimally processed fish
15. Butchery: retail outlets primarily selling fresh meat and meat products
16. Sandwich and sushi shop: food outlets primarily selling ready-to-eat sandwiches, rolls, and/or sushi
17. A liquor store is a retail establishment primarily engaged in the sale of alcoholic beverages, encompassing liquor stores and bottle shops.
18. PUB: mainly involved in selling alcoholic drinks, allowing customers to order and consume alcohol and food on-site. Examples include a pub within a bowling or lawn bowls park, or inside a private gambling club. It can also be located within a park or private club.

Supplementary material 2: Table 2: Association between healthiness of food outlets across regions and Index of Socioeconomic Advantage and Disadvantage (IRSAD) at the suburb and localities level, 2024.

|  |  | Healthy and less healthy | Unhealthy | COR (95% CI) ^a^ | AOR (95% CI) ^b^  Model 1 | AOR (95% CI) ^b^  Model 2 |
| --- | --- | --- | --- | --- | --- | --- |
| Regions | Illawarra | 756 | 678 | **1.62 (1.31, 2.00)** | **1.87 (1.49, 2.34)** |  |
|  | Sapphire Coast | 214 | 190 | **1.61 (1.23, 2.10)** | **1.46 (1.11, 1.92)** |  |
|  | Shoalhaven | 311 | 172 | 1 | 1 |  |
| Index of Socioeconomic Advantage and Disadvantage (IRSAD) | 1 | 386 | 345 | 0.86 (0.67, 1.12) | 0.97 (0.74, 1.27) | 1 |
|  | 2 | 192 | 171 | 0.86 (0.64, 1.16) | 0.90 (0.66, 1.21) | 0.92 (0.71,1.19) |
|  | 3 | 164 | 170 | 1 | 1 | 1.03 (0.79, 1.356) |
|  | 4 | 249 | 170 | **0.61 (0.46, 0.81)** | **0.58 (0.44, 0.77)** | **0.62 (0.48, 0.81)** |
|  | 5 | 290 | 184 | **0.66 (0.50, 0.88)** | **0.60 (0.45, 0.81)** | **0.60 (0.46, 0.77)** |
| Total |  | 1284 | 1037 |  |  |  |

^a^ COR: Crude Odds Ratio; ^b^ AOR: Adjusted Odds Ratio; Model 1: Reference IRSAD quintile 3; Model 2: Reference IRSAD quintile 1
